# Developing Hybrid Machine Learning Models to Determine the Dynamic Modulus (E*) of Asphalt Mixtures Using Parameters in Witczak 1-40D Model: A Comparative Study

**DOI:** 10.3390/ma15051791

**Published:** 2022-02-27

**Authors:** Wenjuan Xu, Xin Huang, Zhengjun Yang, Mengmeng Zhou, Jiandong Huang

**Affiliations:** 1 College of Civil Engineering, Nanjing Forestry University, Nanjing 210037, China; xuwenjuan@jsafc.edu.cn (W.X.); xinhuang@njfu.edu.cn (X.H.); yangzj0511@163.com (Z.Y.); 2 College of Landscape Architecture, Jiangsu Agricultural and Forestry Technical College, Zhenjiang 212400, China; 3 Jurong Traffic and Transport Bureau, Zhenjiang 212400, China; 4 School of Mines, China University of Mining and Technology, Xuzhou 221116, China; ts21020232p21@cumt.edu.cn

**Keywords:** machine learning, dynamic modulus, asphalt mixtures, BAS, hyperparameters

## Abstract

To characterize the dynamic modulus (E*) of the asphalt mixtures more accurately, a comparative study was shown in this paper, combining six ML models (BP, SVM, DT, RF, KNN, and LR) with the novelly developed MBAS (modified BAS, beetle antennae search) algorithm to check the potential to replace the empirical model. The hyperparameter tuning process of the six ML models by the proposed MBAS algorithm showed satisfactory results. The calculation and evaluation process demonstrated fast convergence and significantly lower values of RMSE for the five ML models (BP, SVM, DT, RF, and KNN) to determine the E* of the asphalt mixtures. Comparing the performances of the six ML models in the prediction of the E* by the statistical coefficients and Monte Carlo simulation, the RF model showed the highest accuracy, efficiency, and robustness.

## 1. Introduction

A crucial parameter that distinguishes the flexible pavement performance conditioning on multiple temperatures and loading circumstances in the Mechanistic-Empirical Pavement Design Guide (MEPDG) is the E* (dynamic modulus) of asphalt mixtures [1,2,3]. To achieve an excellent pavement performance and long service life, the precise calculation of the E* is a crucial assignment in the designing process of the pavements [4,5,6,7,8,9,10]. In the previous studies, laboratory-based methods and predictive models were employed to determine the E* to be used in the MEPDG [4,5,6,7,11,12,13,14]. Regarding the laboratory-based methods, it is a typically time-consuming procedure because of the requirements of the notable amount of time to produce the samples, balance the temperature, and conduct the experimental processes [8,15,16,17,18,19,20,21,22,23,24]. Additionally, this complicated experimental test requires trained professional staff and costly devices (e.g., AMPT and SPT). To address the above-mentioned issues, researchers and practitioners around the world have developed several predictive models to determine the E*. Among these, one of the most frequently mentioned is the so-called Witczak models. Another example of these models is the adjusted Hirsch model, which has also been broadly used to determine the E*.

However, although these models have been applied in applications (e.g., in various software and engineering practices), their shortcomings were exposed in recent studies: many researchers challenged the accuracy of these models, particularly at the extreme high and low temperatures [4,6,25,26,27]. Yousefdoost et al. [28] found that the models based on the parameter of viscosity can miscalculate the dynamic modulus, while those models based on the parameter of dynamic shear modulus of the asphalt binder can overvalue the dynamic modulus of the asphalt mixtures. Additionally, Ali et al. [7] and Georgouli et al. [12] discovered that models based on the viscosity can underestimate the dynamic modulus of the asphalt mixtures. Batioja-Alvarez et al. [29] observed that the Hirsh model can underestimate the dynamic modulus of the asphalt mixtures used in the Indiana state. It is worth noting that the inaccuracy of these predicting models originates from the limitations of the regression-based techniques. They are all predicated on linear or nonlinear regression methods. For example, the general form of a regression model to predict the E* of the asphalt mixture is based on the following equation.

(1) $$y=f\left(w_{i}\cdot x_{i}\right)$$

In general, it is difficult to obtain an accurate regression equation by using empirical model, so the regression equation for predicting the E* value of asphalt mixture is limited. Therefore, it is needed to employ more advanced techniques to generate more precise predictive models of E*. Nowadays, machine learning (ML) techniques are widely used to solve different kinds of engineering problems because of their abilities in data processing and optimization. The principal advantage of the ML methods is that they can discover the underlying behavior of a complicated system without needing a previous understanding of the association between the input and the target variable. ML techniques have been applied for predicting the performance of the infrastructural materials and in the realm of pavement engineering. Some of these prominent studies include the discovery of the pavement distress [13], forecast of the international roughness index (IRI) [14], assessment of the segregation of asphalt pavement [15], forecast of the rutting [16], forecast of the fracture energy of asphalt mixtures [17], and calculation of the mechanical characteristics of concretes [18,19]. Regarding the weakness of the regression-based techniques for the prediction of the E*, many scholars have also applied the ML techniques in recent studies, including the M5P model tree algorithm [30], deep learning-based models [31], bagged trees ensemble [32], pre-trained deep learning [33], and gradient decision tree boosting [34].

It should be noted that although the various ML techniques mentioned above were used in predicting E*, there were still some problems that need to be solved: (1) only a limited number of advanced ML algorithms have been used to predict E*, and the reliability and computational efficiency of other advanced ML algorithms, such as random forest (RF), have not been deeply studied in previous studies; (2) the application of ML algorithm always requires proper selection of the hyperparameters, but the optimization ability of beetle antennae search (BAS) for the hyperparameters has not been fully studied in the prediction of asphalt mixture; (3) the existing ML models may have great differences in the calculation results and efficiency when applied to predict the E* of the asphalt mixture, but there is still a lack of systematic and quantitative comparison of these algorithms.

## 2. Research Objective

To address the above-mentioned issues, six ML models, including the back-propagation (BP), decision tree (DT), k-nearest neighbors (KNN), logistic regression (LR), random forest (RF), and support vector machine (SVM), were investigated and compared to predict the E* of asphalt concrete in the present study. These ML algorithms were used to establish the relationships between the design parameters (including Vbeff, Va, ρ200, ρ4, ρ3/8, G*, and δ) and E* of asphalt mixtures, and the BAS algorithm was used to adjust the hyperparameters. This study can be used as a benchmark study for the application of the ML methods to predict the E* of asphalt concrete, which is of great significance in the performance evaluation of flexible pavement in the future. The rest of this article will introduce the dataset descriptions, the application of the artificial intelligence models, the tuning of the hyperparameters, and the summarization of the conclusions.

## 3. Methodology

### 3.1. Overview of the Machines Learning (ML) Models

In the present study, six widely used ML models (including the BPNN, DT, KNN, LR, RF, and SVM models) were employed to predict the E* of the asphalt mixtures using the design factors (Vbeff, Va, ρ200, ρ4, ρ3/8, G*, and δ) from the prediction model in MEPDG. The dataset was collected from the previous studies using the same input and out parameters [35]. In the Superpave mixture design of 16 asphalt mixtures, two types of binders and eight types of aggregate gradations were used. Then, the E* of asphalt mixture was evaluated at three temperatures and three loading frequencies, and 144 laboratory data sets were constructed. It should be noted that the shear dynamic modulus and phase angle were also determined at the same temperature and loading frequency as predicted from the master curves of the binder. Table 1 shows the descriptive statistics of the training and test data sets used for the prediction.

BP neural network is composed of the input layer, output layer, and hidden layer. Each layer of the BP neural network has multiple neurons, the number of which is determined by a specific model. Two or two neurons inside the layer are not interconnected, all neurons are connected with the nearby layer through a one-way connection, and the connected nodes interact with each other through weight [36,37,38,39]. The DT model is a method of approximating the values of discrete functions. This is a typical classification method [40]. The data is processed, rules and decision trees are generated by induction algorithm, and then decision analysis is made on the new data. Decision trees are essentially the process of classifying data through a set of rules. KNN algorithm calculates the distance between the unknown sample and all known samples by judging the category of the unknown sample and taking all known samples as the reference [41,42]. According to the majority-voting law, the KNN algorithm calculates the distance between the unknown sample and all known samples. The unknown samples were classified into the same category as the ones with more categories in the K nearest neighbor samples. LR algorithm can be used for classification and prediction. It can predict the probability of occurrence of future results based on the performance of historical data [43,44]. The goal of linear regression fitting is to keep the data points on a straight line as much as possible, while the goal of LR is to try to place the points of different categories on both sides of the straight line. RF is a highly accurate prediction for the dataset but is not easy to overfit. Additionally, it can rank the importance of the resulting variables and can process both discrete and continuous data, and does not need to be normalized [45,46,47]. SVM is a kind of generalized linear classifier based on supervised learning. The decision boundary is the maximum boundary hyperplane of the learning sample [17]. A more detailed description of these six ML models can be found in the previous studies [48,49,50].

### 3.2. Modified Beetle Antennae Search (MBAS)

#### 3.2.1. Beetle Antennae Search (BAS) Algorithm

BAS algorithm is a recently proposed heuristic optimization algorithm, which is inspired by the search behavior of beetles [33,40].

To determine the global optimality regarding a multidimensional space, Equation (2) should be employed as described below:

(2) $$\frac{Minimize}{Maximize}f\left(x\right),\;x={\left[x_{1},x_{2},\;\cdots ,x_{N}\right]}^{T}$$

The following equation can be used to define the beetle’s searching behavior.

(3) $$b=\frac{rnd\left(k,1\right)}{\|rnd\left(k,1)\right\|}$$

(4) $$x_{r}=x^{i}+d^{i}b$$

(5) $$x_{l}=x^{i}-d^{i}b$$

The following equation can be used to define the detecting behavior of the beetle.

(6) $$x^{i+1}=x^{i}+\delta^{i}b\cdot sign\left(f\left(x_{r}\right)-f\left(x_{l}\right)\right)$$

The following equation can be used to describe the step size of the updating equation.

(7) $$\delta^{i+1}=\eta \delta^{i}$$

where η is the coefficient of step size to characterize the attenuation.

#### 3.2.2. MBAS Algorithm

For traditional BAS, the beetle’s step size is constant or decreases with each iteration. There are problems with this step-size strategy. If the given step size is not big enough, the BAS algorithm may converge slowly or fall into a local optimal state. Therefore, Levy flight and self-inertial weight were used to adjust BAS step size in this study. To improve the search efficiency, this paper named the improved the BAS to the MBAS. The following objectives can be achieved: (i) the step size can be adjusted quickly based on the value of the current fitness, and the adaptive weight can be used to reduce the oscillation; (ii) Levy flight was used to improve the step size randomly if the BAS fell into the state of local optimal.

(i). Levy flight

During the calculation process of the traditional BAS algorithm, the step size is kept constant or decreasing in one iteration, which leads to the BAS algorithm falling into the state of local optimal easily. To solve this problem, Levy flight was adopted to modify the step size of the BAS algorithm. From the early research results, Levy flight is useful to adjust step size [51,52,53]. In this study, if the BAS algorithm is in a locally optimal state, the step size of the beetle can be increased by using Equation (8).

(8) $$\delta^{\left(i\right)}=\alpha \left|Levy\right|⊗\delta^{\left(i-1\right)}$$

in which *α* is a parameter to describe the random value between 0 and 1. ⊗ represents multiplication by item; |Levy| is a variance of infinite Levy distribution, and the infinite is described as $Levy\sim u=t^{-\lambda },(1<\lambda \leq 3)$. The Levy flight can be triggered as

(9) $$\left|f^{\left(i\right)}-f^{\left(i-1\right)}\right|<\mu \left(f_{w}-f_{b}\right)$$

where *μ* is the parameter, which is determined as 10^−5^ in the present study.

(ii). Self-adaptive inertia weight

In the present study, the adaptive inertia weights are applied to a monotone reduction equation, as described by the following equation.

(10) $$\delta^{i+1}=\eta^{i}\times \delta^{i}$$

where $\delta^{i}$ is the step of the current position; $\eta^{i}$ represents the adaptive inertia weight, which is determined by:

(11) $$\eta^{i}=\left(1-\alpha \right)0.95+\alpha \frac{f_{w}^{i}-f^{i}}{f_{w}^{i}-f_{b}^{i}}$$

where $f^{i}$ is the fitting equation of the current position; $f_{b}^{i}$ is the best fit value. $f_{w}^{i}$ is the worst fit value; *α* is the tradeoff between two parameters: the first item ((1−α)0.95) is the inertia weight, the second ($\alpha \frac{f_{w}^{i}-f^{i}}{f_{w}^{i}-f_{b}^{i}}$) is an adaptive feature. In this study, *α* was determined to be 0.2 after modification of preliminary trial calculation.

### 3.3. Methods for the Evaluation and Calibration

#### 3.3.1. Determine the Predicting Performance

The parameter used to evaluate and calibrate the proposed model was Root Mean Square Error (RMSE) [44], which employs the following equation to calculate the difference between the predicted value and the actual measured value.

(12) $$RMSE=\sqrt{\frac{1}{n}\overset{n}{\underset{i=1}{\sum }}{\left(y_{i}^{*}-y_{i}\right)}^{2}}$$

where $y_{i}^{*}$ and $y_{i}$ represent predicted value and actual measured value, respectively. *n* indicates the number of data samples. Another parameter used to evaluate predictive performance is the correlation coefficient (*R*), which is determined by the correlation between predicted and actual values, as shown in the following equation [54].

(13) $$R=\frac{\sum_{i=1}^{n}\left(y_{i}^{*}-\bar{y^{*}}\right)\left(y_{i}-\bar{y}\right)}{\sqrt{\sum_{i=1}^{n}{\left(y_{i}^{*}-\bar{y^{*}}\right)}^{2}}\sqrt{\sum_{i=1}^{n}{\left(y_{i}-\bar{y}\right)}^{2}}}$$

where $\bar{y^{*}}$ and $\bar{y}$ are the mean value for the predicted ones and actual ones, respectively.

#### 3.3.2. K-Fold Cross-Validation

**Figure 1:**
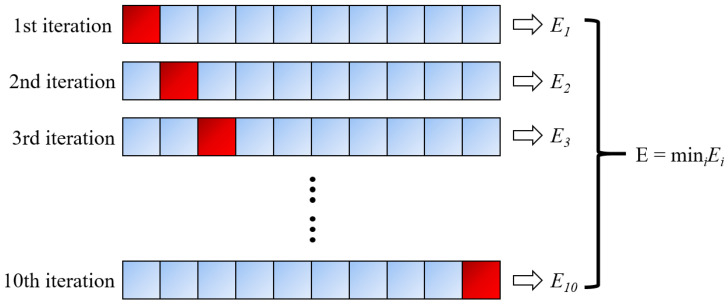
Ten-fold cross-validation (CV) process.

For the correction of the regression model, the early studies adopted the simple substitution method, support method, retention method, and Bootstrap method [55,56,57]. Regarding the approaches employed to validate the training data, the most widely used k-fold cross-validation (CV) was employed in the present research [58]. Specifically, k is specified as 10, taking into account the recommendations of earlier studies [59]. Therefore, the training data set is split by a factor of 10 during hyperparameter tuning. The algorithm has been trained for nine times and verified for 10 times. This process should be repeated 10 times, each time verifying with a different fold. The final result is determined to be the result with the smallest error in a single fold. Figure 1 summarizes the CV process above.

### 3.4. Hyperparameter Tuning

**Figure 2:**
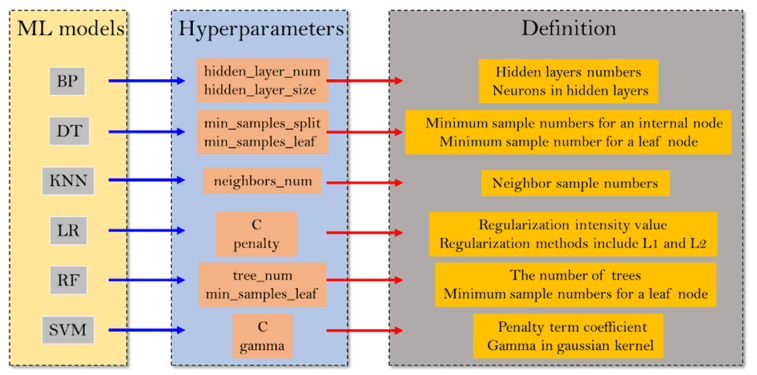
Hyperparameters of the six ML algorithms.

Figure 2 gives the hyperparameters of the six ML models that were tuned by the MBAS.

To determine the effect of MBAS algorithm, this study uses the formula as a Benchmark function to find the trajectory and contraction curve of the MBAS algorithm.

(14) $$f\left(x\right)=\sum_{i=1}^{n}-x_{i}\sin\left(\sqrt{\left|x_{i}\right|}\right)$$

Equation (14) shows that as x goes to infinity, y goes to minus infinity. In other words, the smaller the y value, the better the performance of the optimization algorithm. According to the above description, the hyperparameters of the 6 ML modelS were adjusted using the 10-fold CV and MBAS. After 10 folds, 10 hyperparametric data sets can be obtained. For these 10 subsets, the MBA algorithm needs to search the optimal hyperparameters of 9 subsets and evaluate the RMSE of 1 subset. After convergence, the optimal hyperparameter of the first iteration can be determined. Repeat this process 10 times according to the requirement of 10 CV, and verify the different sets each time. Through the above steps, 10 RMSE values can be obtained, and the smallest RMSE is selected to correspond to the optimized hyperparameter. Finally, the predictive performance evaluation test data set of the six ML models were independent of the training data set (30% of the total data set). Therefore, the test data set is only used to evaluate predictive performance. It should be noted that minimum overfitting occurs once the model has a good fit for the training and test data sets.

## 4. Results and Discussion

### 4.1. Results of the Hyperparameter Tuning

As mentioned above, the proposed MBAS algorithm was used in this study to tune the hyperparameters of the six ML models, and the optimized hyperparameters were determined for verification according to the hyperparameter set of the minimum RMSE value in the data set. Table 2 gives the optimum hyperparameters of the six ML models.

**Figure 3:**
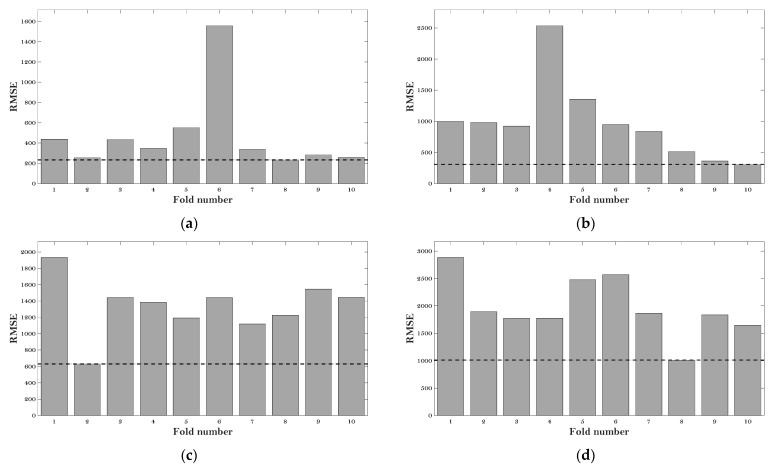

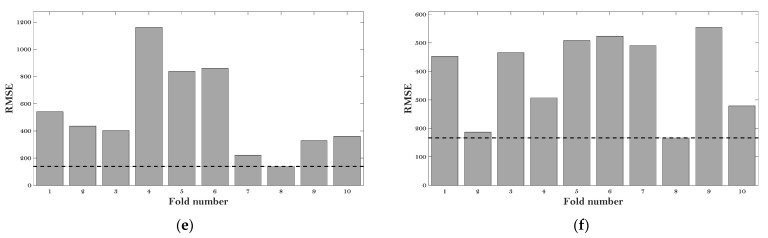
RMSE results of the hyperparameter tuning: (**a**) BP; (**b**) DT; (**c**) KNN; (**d**) LR; (**e**) RF; and (**f**) SVM.

Figure 3 gives the RMSE values of the six ML models at the various fold number.

It can be observed that the minimum values of RMSE (which are 220 MPa, 300 MPa, 625 MPa, 1000 MPa, 175 MPa, and 185 MPa) can be obtained at the fold number of 8, 10, 2, 8, 8, and 8 concerning the BP, DT, KNN, LR, RF, and SVM models, respectively. Therefore, the hyperparameters at these fold numbers were employed for these six algorithms to evaluate the E* of the asphalt mixtures.

**Figure 4:**
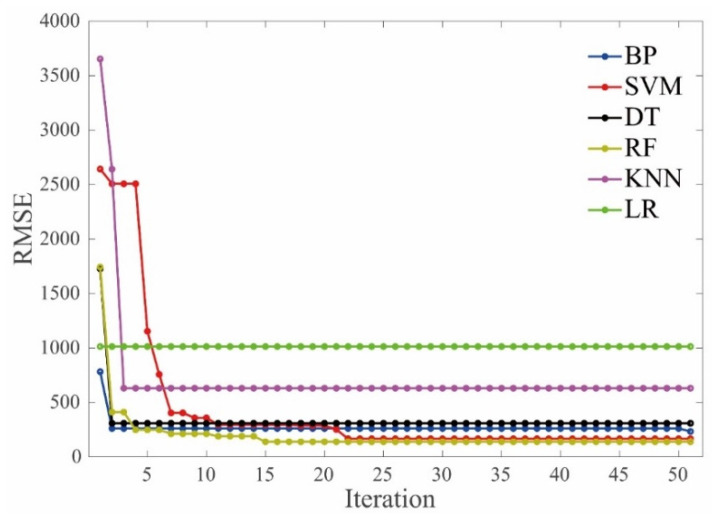
Iteration vs. RMSE values.

Figure 4 gives the average values of RMSE versus iteration during the hyperparameter tuning process. It should be noted that no further decrease of the RMSE values was observed after 50 iterations for all the ML algorithms.

It can be observed that different ML models showed different evolution modes regarding the RMSE values and convergence speed. The RMSE value of the LR model tended to be stable from the initial iteration, indicating that the influence of the hyperparameters on the performance of the LR algorithm has no obvious effect on the dataset of the E*. The convergence speed of the RF and SVM models was slightly slower than the others when they were used to evaluate the E* of the asphalt mixtures. The RMSE values of the RF and SVM models tended to convergent at the iteration of 15 and 22, respectively. The RMSE values of the KNN and SVM models decreased more quickly compared to the other models when the BAS was employed to tune the hyperparameters, indicating that the BAS can tune the KNN and SVM algorithms more efficiently to evaluate the dynamic modulus of the asphalt mixtures. In terms of the overall effect, the BAS algorithm can efficiently find the optimized hyperparameters of these ML models.

Regarding the accuracy of the tuning hyperparameters process, it can be seen that LR and KNN models behaved the worst, indicated by the largest RMSE values (which are 1015 MPa and 520 MPa, respectively) after the convergence. The BP, SVM, DT, and RF models showed relatively small values of RMSE, indicating the BAS can tune the hyperparameters of these four algorithms reliably.

### 4.2. Comparison of the Predictive Performance

**Figure 5:**
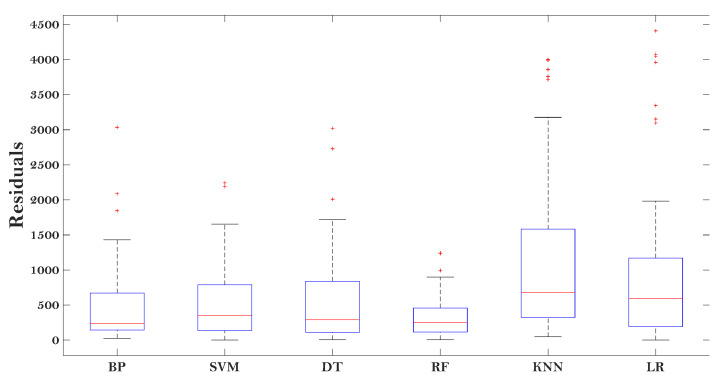
Residual results of various predictive models.

Figure 5 gives the results (which are described by the box-plot) of the residual between the predicted E* and actual E* of the asphalt mixtures.

It can be seen that the RF performed the best among all the models, indicated by the minimum residual value. The KNN and LR models showed large residuals, demonstrating that they may not be suitable to evaluate the E* of the asphalt mixtures. The other three models (BP, SVM, and DT) showed moderate performance in predicting the E* of the asphalt mixtures, with certain accuracy. The results in Figure 5 indicate enough accuracy determined from the proposed models (RF, BP, SVM, and DT) modified by the MBAS algorithm.

**Figure 6:**
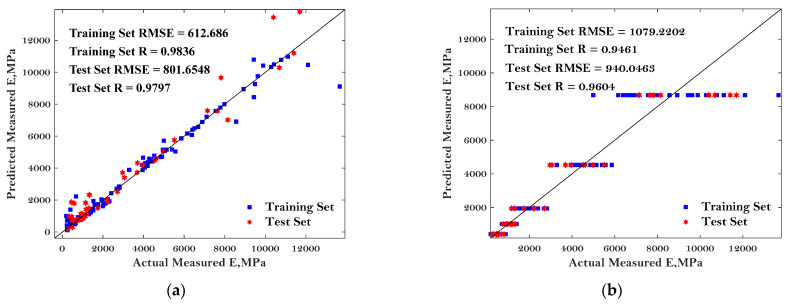

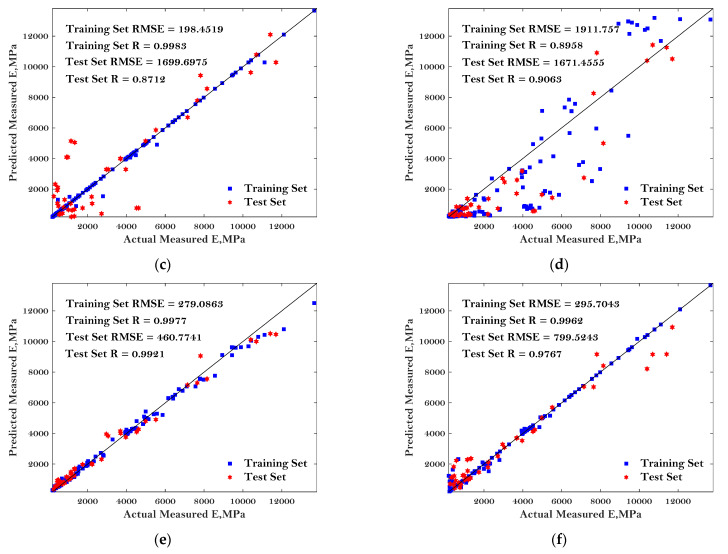
Comparison of the predicted dynamic modulus and measured dynamic modulus: (**a**) BP; (**b**) LR; (**c**) DT; (**d**) KNN; (**e**) RF; and (**f**) SVM.

To more comprehensively evaluate the performance of these six ML algorithms in predicting the E* of asphalt mixture, the predicted dynamic modulus of the asphalt mixture, the actual measured dynamic modulus of the asphalt mixture, as well as the ‘1:1’ curve were plotted, as shown in Figure 6.

The predictive performance of the six models was evaluated by the two most commonly used parameters: RMSE and R, as mentioned in the previous chapter. Better predictive performance of the BP, DT, RF, and SVM models were observed compared to the others except for the few noise points. Additionally, the good predictive performance of these four models can be indicated by the lower value of RMSE and higher value of R. The results in Figure 6 can also indicate enough accuracy determined from the proposed MBAS algorithm. Regarding the RF algorithm, the best agreements can be observed between the comparison results, with the correlation coefficients of 0.9921 and 0.9977 for the testing dataset and training dataset, respectively. The worst-performing models were LR and KNN with the highest values of RMSE and lowest values of R, indicating that these baseline algorithms may not suitable to predict the E* of asphalt mixtures.

**Figure 7:**
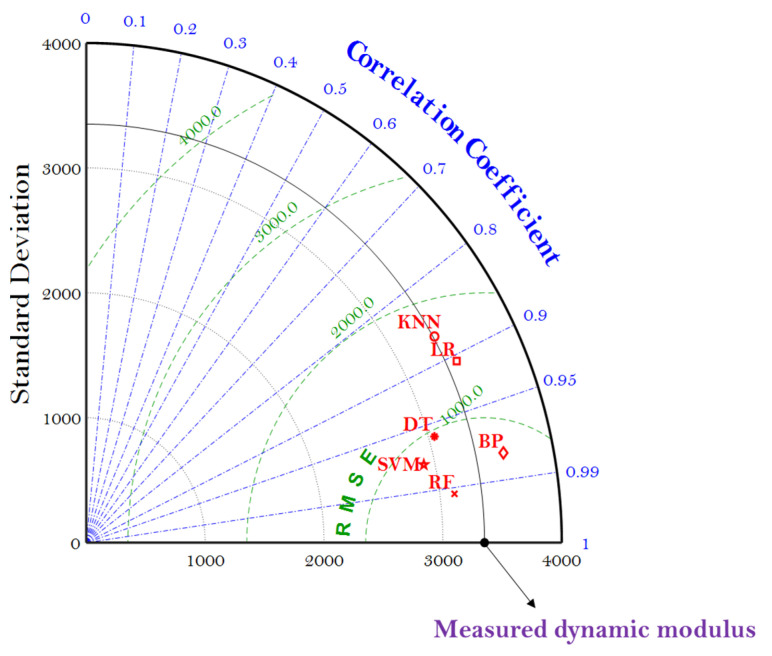
Taylor diagram of the six ML models using the MBAS algorithm.

The reliability characteristics of these ML models can also be graphically represented by the Taylor diagram (which is generally used to evaluate the accuracy of the ML model), including the information of the R, standard deviation (SD), and RMSE. In general, the scatters in the Taylor diagram represent various ML models, the radial line represents the R, the horizontal and vertical axes represent the SD, and the dashed line represents the RMSE. Taylor diagram is a modification from the previous use of the scatter diagram, which can only present two indicators to represent the accuracy of the ML model. Figure 7 gives the Taylor diagram of the six ML models as they were employed to predict the E* of the asphalt mixtures.

It can be seen that SVM, RF, DT, and BP models are the closest to the measured dynamic modulus with relatively higher R and lower RMSE, indicating the MBAS algorithm can tune the hyperparameters of these four ML models well to predict the E* of the asphalt mixtures. It should be noted that the RF model exhibited the lowest values of RMSE and SD, as well as the highest value of R, performing best in the prediction.

To clarify the randomness characteristics of different ML models, this study employed the widely used Monte Carlo simulation [60], where a random combination of input variables that fit a certain probability distribution is selected and the ML models are used to predict the output. The uncertainty of the input variable is propagated to the output by Monte Carlo simulation.

**Figure 8:**
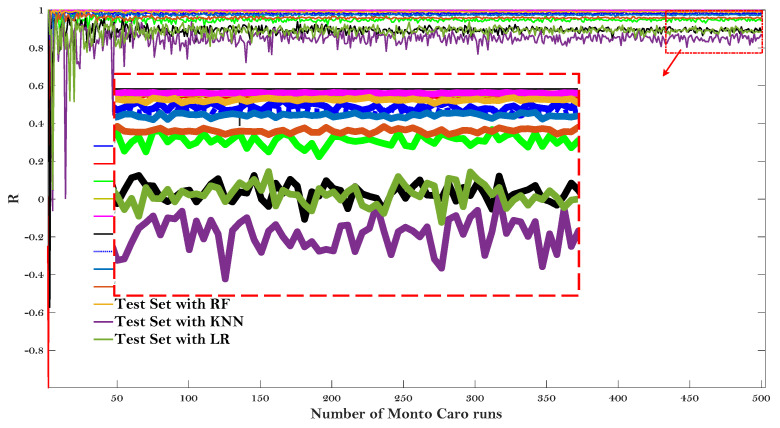
Monte Carlo simulation (Number of Monto Carlo runs vs. value of R).

**Figure 9:**
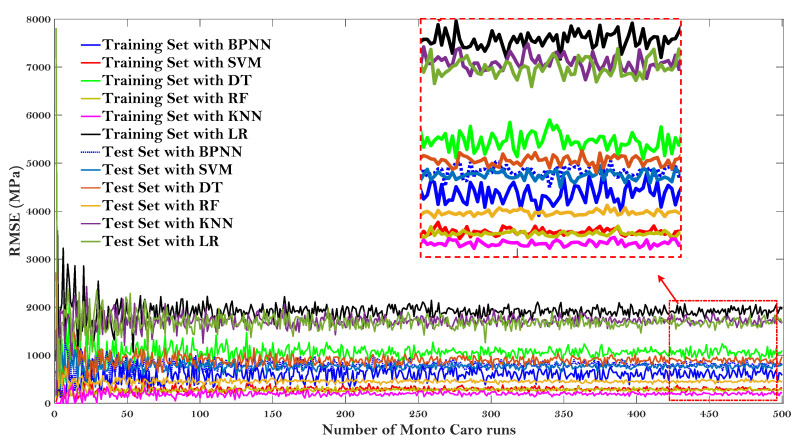
Monte Carlo simulation (Number of Monto Carlo runs vs. value of RMSE).

Figure 8 and Figure 9 give the results of the Monte Carlo simulation (Number of Monto Carlo runs vs. values of R and RMSE, respectively).

All the values of R and RMSE tended to converge as the Monto Carlo runs 500 times. LR model showed the lower R and higher RMSE on both the test and training dataset after the simulation results converged, once again proving that this baseline model may not be suitable to predict the E* of the asphalt mixtures. It is worth noting that the KNN model can obtain the lowest value of R for the test dataset, but can obtain almost the highest value of R for the training set (for the values of RMSE, it also obtain the highest and lowest RMSE, respectively). The same results were presented for RMSE values and were not repeated here. It indicated that the KNN model is not very stable to predict the dynamic modulus of the asphalt mixture. Additionally, it can be observed that the RF model obtained almost the highest value of R and lowest value of RMSE regarding both the testing and training dataset, demonstrating that the RF model is more stable to predict the dynamic modulus of the asphalt mixtures.

## 5. Conclusions

In this study, six ML models (BP, SVM, DT, RF, KNN, and LR) were implemented and compared using the newly developed Modified BAS (MBAS) algorithm to replace the mathematical regression model in MEPDG. The conclusions can be summarized as follows.

The BAS algorithm is modified by Levy flight and inertia weight, which avoids premature convergence to local optimum and improves search efficiency compared with the traditional BAS algorithm. The calculation results show that the five ML models (BP, SVM, DT, RF, and KNN) for evaluating the E* of the asphalt mixtures have fast convergence speed and low RMSE value, which proves the effectiveness and accuracy of the MBAS algorithm. However, for dynamic modulus data sets, hyperparameters have no significant effect on the performance of LR models. The comparison results show that the MBAS algorithm can adjust the hyperparameters of SVM and KNN more effectively.

Comparing the performances of the six ML models in the prediction of asphalt mixture’s dynamic modulus by the statistical coefficients (values of R, RMSE, and SD) and Monte Carlo simulation, the RF model showed the highest accuracy, efficiency, and robustness.

In future studies, more experimental tests will be conducted and updated databases will be continuously collected to improve the applicability of the proposed model. The application of the models on a different set of asphalt concretes will be conducted to evaluate whether the model can be generally used in asphaltic materials.

## Figures and Tables

**Table 1:** Descriptive statistics of the training and test data sets used for the prediction.

Dataset	Variables	Mean	STD	Skewness
Training dataset (the number of data points is 100)	E*	3532.0	3272.3	1.0
	Vbeff	3.5	0.4	−0.4
	Va	35.7	2.7	−0.3
	ρ200	16.6	4.3	0.4
	ρ4	6.9	0.1	−0.3
	ρ3/8	10.5	0.7	−0.3
	G*	2.8	5.2	2.1
	δ	59.1	12.1	−0.2
Testing dataset (the number of data points is 44)	E*	3557.9	3530.2	1.2
	Vbeff	3.7	0.6	−1.3
	Va	36.2	3.0	0.4
	ρ200	17.8	2.7	1.4
	ρ4	6.9	0.2	−0.2
	ρ3/8	10.5	0.7	−1.2
	G*	2.8	5.1	2.2
	δ	57.5	12.2	−0.1

**Table 2:** Optimum hyper-parameters of the six ML algorithms.

Models	Hyper-Parameters	Empirical Scope	Initial Value	Result
BP	hidden layer num	[1,4]	{1,2,3,4}	3
	hidden layer size	[1,20]	30, (20,10), (20,10,10), (10,10,10,10)	(8,6,8)
DT	min samples split	[1,10]	25	4
	min samples leaf	[2,10]	50	8
KNN	neighbors num	[1,10]	30	1
LR	tol	[1 × 10^−5^–1 × 10^−3^]	1	1.18 × 10^−4^
	c inverse	0.1–10	10	220
RF	tree num	[1,10]	40	7
	min samples leaf	[1,10]	40	1
SVM	C	[0.1,1000]	16	4
	gamma	[0.001,100]	16	4.8 × 10^−3^

## Data Availability

The data presented in this study are available on request from the corresponding author.
